# Supplementary material for: Structure of the Signal Transduction Domain in Second-Generation CAR Regulates the Input Efficiency of CAR Signals
Source: Int J Mol Sci. 2021 Mar 1;22(5):2476. doi: 10.3390/ijms22052476 (PMC7957710; doi:10.3390/ijms22052476)
Supplement: Supplementary file 1 [file ijms-22-02476-s001.pdf]

International Journal of
Molecular Sciences

MDPI

Supplementary Materials

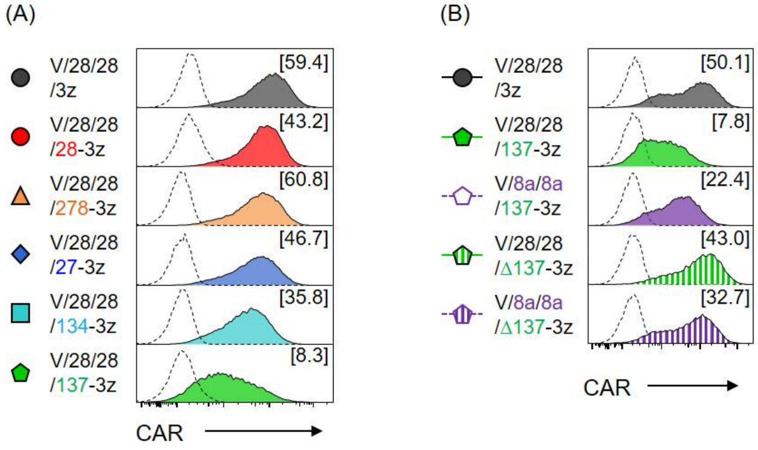

Supplementary Figure S1. The analysis of the CAR expression on mouse CD8⁺ T cells, shown in Figure 1. (A) CAR expression on T cells after Rv transduction (day 0) was analyzed by flow cytometry using anti-HA-tag (solid color histograms) or isotype control antibodies (dashed white histograms). The expression level of each CAR shown in brackets was calculated from the ratio of GMFI when stained with the anti-HA-tag mAb to GMFI when stained with the isotype control antibody. The data are representative of at least two independent experiments.

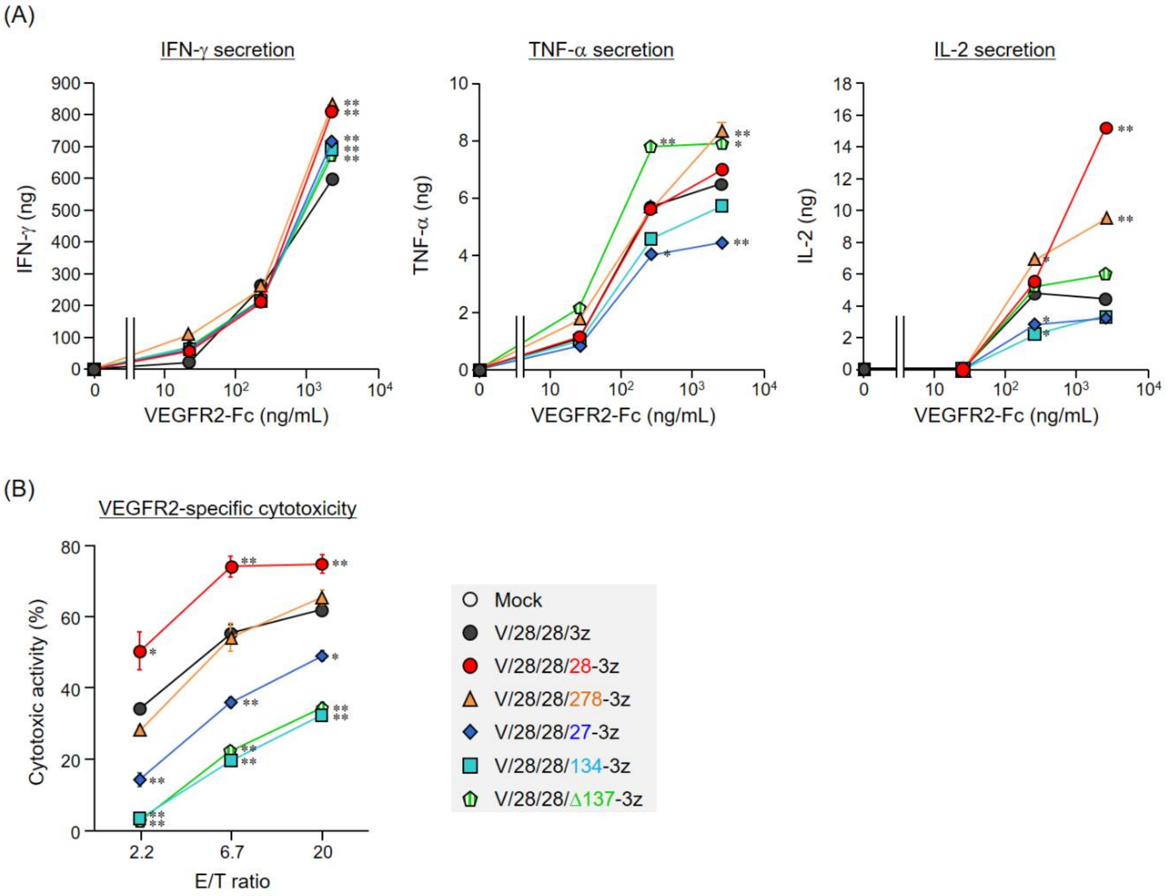

Supplementary Figure S2. Cytokine production and cytotoxic activity of mouse T cells expressing second-generation CARs, shown in Figure 2. (A) CAR-T cells cultured for 4 days after Rv transduction were stimulated with VEGFR2-Fc (0–2000 ng/mL) for 24 h. The amount of IFN-γ, TNF-α, or IL-2 secreted by CAR-T cells after 24 h stimulation was determined by ELISA. The data are shown as the mean ± SD of triplicates, and are representative of two independent experiments. Statistical analysis was performed using the Tukey's test: $*p < 0.05$ and $**p < 0.01$. (B) CAR-T cells were cultured for 4 days after Rv transduction, and then were co-cultured with EL4 cells and VEGFR2$^+$ EL4 cells at an indicated E/T ratio for 18 h. Then, the numbers of EL4 cells and VEGFR2$^+$ EL4 cells in the wells were evaluated by flow cytometry. The cytotoxic activity against VEGFR2$^+$ EL4 cells was calculated from the ratio between the numbers of VEGFR2$^+$ EL4 cells to that of EL4 cells. The data are shown the mean ± SD of triplicate and are representative of two individual experiments. Statistical analysis was performed using the Tukey's test with second-generation CARs versus first-generation CAR (V/28/28/3z): $*p < 0.05$ and $**p < 0.01$.

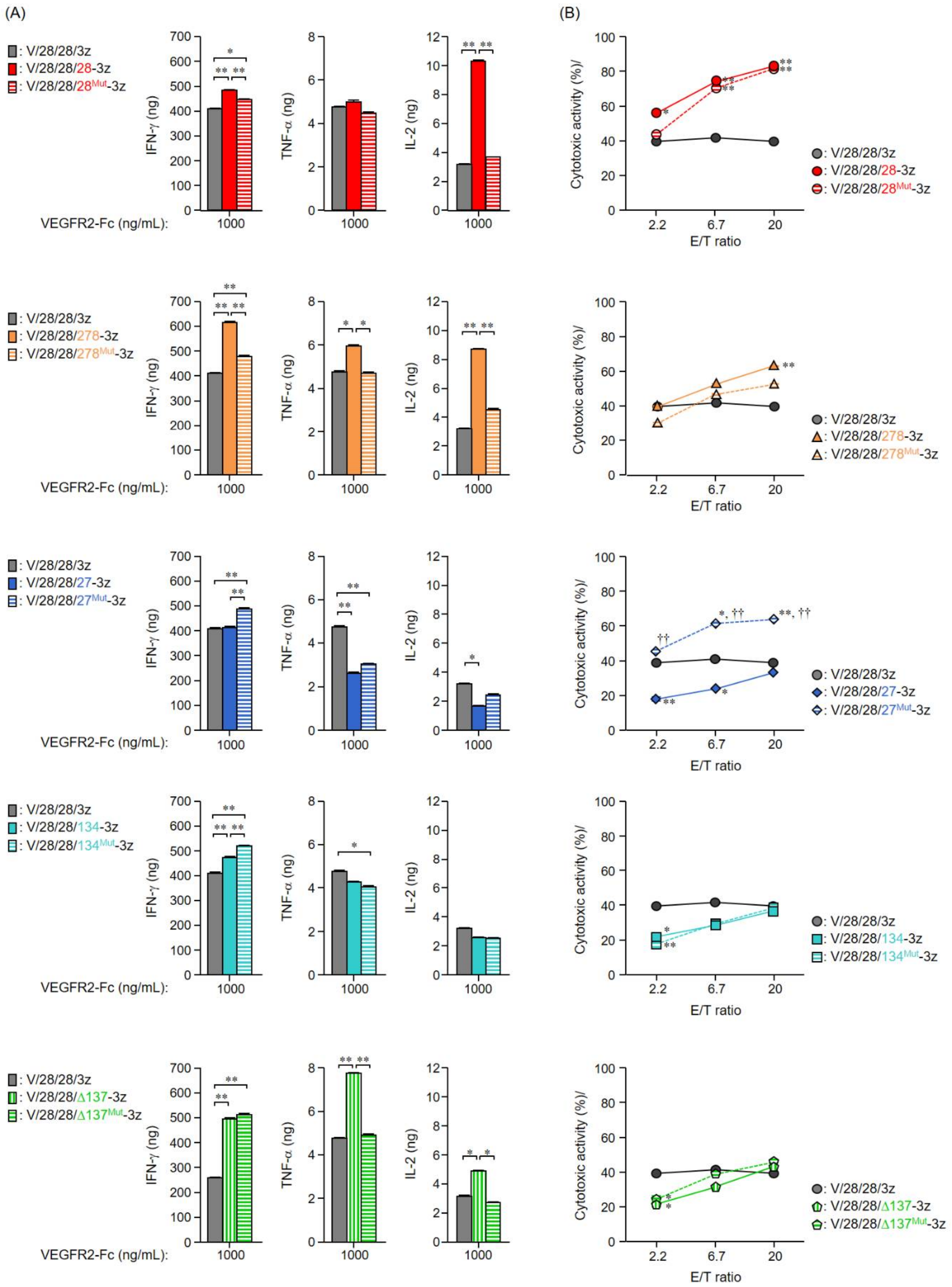

Supplementary Figure S3. Cytokine production and cytotoxic activity of mouse T cells expressing second-generation CARs that cannot input the 2nd STD signals, shown in Figure 3. (A) CAR-T cells cultured for 4 days after Rv transduction were stimulated with VEGFR2-Fc (1000 ng/mL) for 24 h. The amount of IFN-γ, TNF-α, or IL-2 secreted by CAR-T cells after 24 h stimulation was determined by ELISA. The data are shown as the mean ± SD of triplicates, and are representative of two independent experiments. Statistical analysis was performed using the Tukey's test: *$p < 0.05$ and **$p < 0.01$. (B) CAR-T cells were cultured for 4 days after Rv transduction, and then were co-cultured with EL4 cells

and VEGFR2$^+$ EL4 cells at an indicated E/T ratio for 18 h. Then, the numbers of EL4 cells and VEGFR2$^+$ EL4 cells in the wells were evaluated by flow cytometry. The cytotoxic activity against VEGFR2$^+$ EL4 cells was calculated from the ratio between the numbers of VEGFR2$^+$ EL4 cells to that of EL4 cells. The data are shown the mean ± SD of triplicate and are representative of two individual experiments. Statistical analysis was performed using the Tukey's test with second-generation CARs versus first-generation CAR (V/28/28/3z): *$p < 0.05$ and **$p < 0.01$; second-generation CARs deficient in the 2nd STD signal versus unmodified second-generation CARs: ††$p < 0.01$.

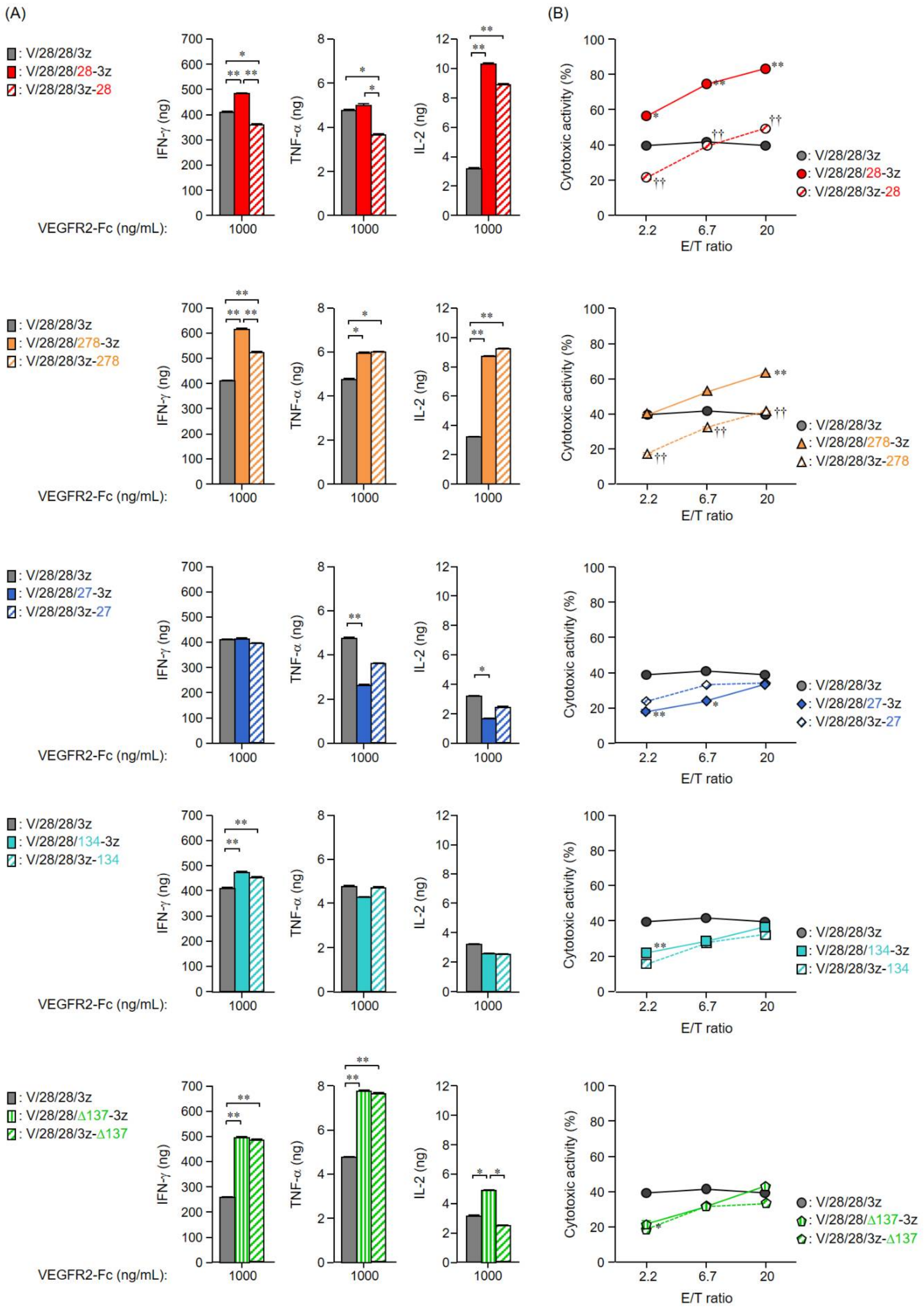

Supplementary Figure S4. Cytokine production and cytotoxic activity of mouse T cells expressing second-generation CARs with reordered STDs, shown in Figure 4. (A) CAR-T cells cultured for 4 days after Rv transduction were stimulated with VEGFR2-Fc (1000 ng/mL) for 24 h. The amount of IFN-γ, TNF-α, or IL-2 secreted by CAR-T cells after 24 h stimulation was determined by ELISA. The data are shown as the mean ± SD of triplicates, and are representative of two independent experiments.

Statistical analysis was performed using the Tukey's test: *$p < 0.05$ and **$p < 0.01$. (B) CAR-T cells were cultured for 4 days after Rv transduction, and then were co-cultured with EL4 cells and VEGFR2$^+$ EL4 cells at an indicated E/T ratio for 18 h. Then, the numbers of EL4 cells and VEGFR2$^+$ EL4 cells in the wells were evaluated by flow cytometry. The cytotoxic activity against VEGFR2$^+$ EL4 cells was calculated from the ratio between the numbers of VEGFR2$^+$ EL4 cells to that of EL4 cells. The data are shown the mean ± SD of triplicate and are representative of two individual experiments. Statistical analysis was performed using the Tukey's test with second-generation CARs versus first-generation CAR (V/28/28/3z): *$p < 0.05$ and **$p < 0.01$; second-generation CARs with reordered STDs versus unmodified second-generation CARs: ††$p < 0.01$.

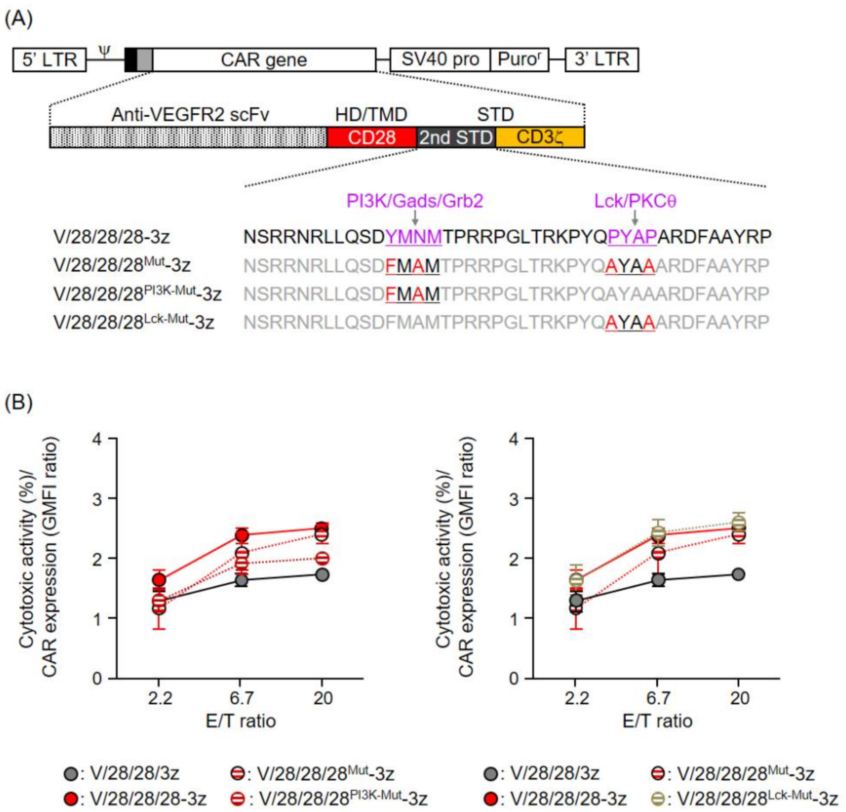

Supplementary Figure S5. Functional characteristics of mouse T cells expressing second-generation CARs that cannot input CD28-STD signals. (A) Amino acid sequence of CD28-STD in second-generation V/28/28/28-3z. Adaptor molecules (PI3K/Gads/Grb2 and Lck/PKCθ) that bind to the CD28-STD in V/28/28/28-3z and their binding regions are shown in magenta. Amino acid mutation sites in CD28-STD signal input-deficient CARs (V/28/28/28^{Mut}-3z, V/28/28/28$^{PI3K\text{-}Mut}$-3z, and V/28/28/28$^{Lck\text{-}Mut}$-3z) are shown in red. (B) Relationship between cytotoxicity at indicated E/T ratio and CAR expression level in each CAR-T cells. CAR-T cells cultured for 4 days after Rv transduction, were co-cultured with EL4 cells and VEGFR2$^+$ EL4 cells at an indicated E/T ratio for 18 h. Then, the numbers of EL4 cells and VEGFR2$^+$ EL4 cells in the wells was evaluated by flow cytometer. The cytotoxic activity against VEGFR2$^+$ EL4 cells was calculated from the ratio between the numbers of VEGFR2$^+$ EL4 cells to that of EL4 cells. The data shows the mean ± SD of triplicate.

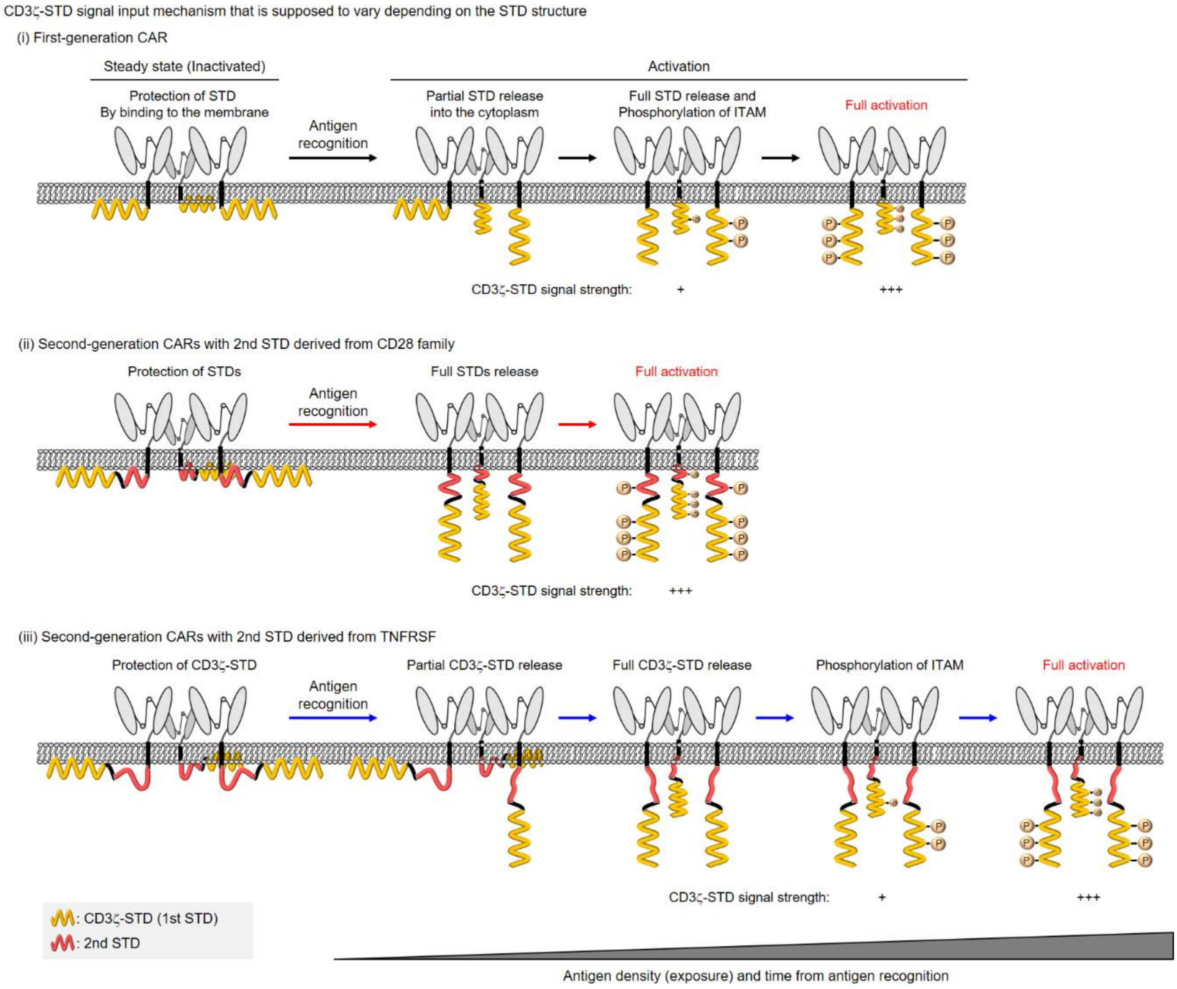

Supplementary Figure S6. Hypothesis on the signal input mechanism of second-generation CARs. The illustrations show the hypothesized mechanism of CD3ζ-STD signaling in first-generation CARs (i) and second-generation CARs with a 2nd STD derived from the CD28 family (ii) or TNFRSF (iii), based on the findings in this study. CD3ζ-STD in the steady state is lined by the plasma membrane. Binding of ARD to the target antigen causes a mechanical change in the structure of CAR, which releases CD3ζ-STD into the cytoplasm. ITAM in the CD3ζ-STD exposed in the cell is phosphorylated by Lck and enters the T cell activation signal. (ii) Like CD3ζ, CD28 and CD278 also keep signal input off by binding intracellular regions to the membrane in the steady state. It is inferred that second-generation CARs with CD28 family have a higher efficiency of intracellular exposure of STDs after antigen recognition and a faster rate of Lck-mediated phosphorylation of ITAM in CD3ζ-STD. (iii) TNFRSF transmits signals via TRAF; the intracellular region is not bound to the membrane. Second-generation CARs with TNFRSF-STD are inferred to have a lower efficiency of STDs exposure after antigen recognition and a slower rate of Lck-mediated phosphorylation of ITAM within CD3ζ-STD.

Murine CD137 STD:	S V L K W I R K K F P H I F K Q P F K K T T G A A <u>Q E E D</u> A C S C R C P <u>Q E E E</u> G G G G G Y E L
Human CD137 STD:	K R G R K K L L Y I F K Q P F M R P V Q T T <u>Q E E D</u> G C S C R F P <u>E E E E</u> G G C E L

Supplementary Table S1. Amino acid sequence of mouse and human CD137 intracellular domain. TRAFs binding sites in the sequence are underlined.
